# Supplementary material for: Comparative genomics of high grade neuroendocrine carcinoma of the cervix
Source: PLoS One. 2020 Jun 16;15(6):e0234505. doi: 10.1371/journal.pone.0234505 (PMC7297329; doi:10.1371/journal.pone.0234505)
Supplement: Attachment — Submitted filename: Plos One-03042020.docx [file pone.0234505.s002.docx]

Dear Dr. Xie,

Please find below my review of the manuscript entitled “**Comparative genomics of high-grade neuroendocrine carcinoma of the cervix**” submitted by Robert Tyler Hillman et al. The manuscript No. is PONE-D-20-05313.

Robert Tyler Hillman and other co-authors intended to comprehensively investigate the genomics landscape of high-grade neuroendocrine carcinoma of the cervix (NECC) through multiple algorithms of computational biology. NECC is a rare type of malignant cervical cancers with worse overall survival compared to other types of cervical cancer. Development of an effective treatment is critical to improve patients’ life quality. The study is meaningful, but the less support for its clinical translational significance.

The topic of this manuscript has potential for a clinical translational apply, but the quality of the studies in it looks a little bit modest and superficial. In the spirit of making the work better, I sincerely provide my advices to the authors.

First, whole exome sequencing and multiple bioinformatics analysis were used to identify the genomics of NECC in this manuscript. The identified mutations in NECC represented the genomic changes under tumor condition, these may have implications in NECC development and therapy response, a deep literature review and discussion will make a more clarity of the Importance of the mutations.

Second, the authors identified and compared mutations and CNVs in NECC and matched normal tissue, the sample size is quite small, the frequency of some mutations was relatively low, the comparison may be invalid. Meanwhile, comparative analysis was performed to explore the difference between NECC and other types of cervical cancers and extra-cervical small cell carcinomas, the clinical stage should be moderately matched.

Third, as the authors declared, the sample size is low, and the clinical implication was limited, although the authors try to extend the results to suggest some potential therapies that patients may benefit. Further validation should be conducted.

In addition, the abstract could be more summative and concise.

In summary, the bioinformatics analysis is ok, but the sample size is relatively small, so the clinical translational significance of the findings may be limited. If a bigger sample size and more clinical data support for the conclusion, the paper will be good to accept.

Please feel free to contact me if you require further details.

Hua Jiang, PhD

Postdoctoral fellow-Pediatrics and Immunology

Indiana University School of Medicine

1044 W. Walnut Street, Room R4-440

Indianapolis, IN, 46202

Phone: 317-531-6947

E-mail: jianghu@iu.edu
